# Two Lignan Glycosides from *Albizia julibrissin* Durazz. Noncompetitively Inhibit Serotonin Transporter

**DOI:** 10.3390/ph15030344

**Published:** 2022-03-11

**Authors:** Bishan Huang, Hanhe Liu, Yingyao Wu, Chan Li, Qingfa Tang, Yuan-Wei Zhang

**Affiliations:** 1 School of Life Sciences, Guangzhou University, Guangzhou 510006, China; 2111914032@e.gzhu.edu.cn (B.H.); jokerliuhh@e.gzhu.edu.cn (H.L.); 2112014072@e.gzhu.edu.cn (Y.W.); lichan@gzhu.edu.cn (C.L.); 2 School of Traditional Chinese Medicine, Southern Medical University, Guangzhou 510515, China; tangqf@smu.edu.cn; 3 Guangdong Provincial Key Laboratory of Chinese Medicine Pharmaceutics, Guangzhou 510515, China

**Keywords:** *Albizia julibrissin* Durazz., antidepressants, serotonin transporter, monoamine transporters, mechanism of action, noncompetitive inhibition

## Abstract

*Albizia julibrissin* Durazz. is one of the most common herbs used for depression and anxiety treatment, but its molecular basis and mechanism of action as an antidepressant or anxiolytic drug are not understood. In this study, we separated and identified two lignan glycosides that inhibit serotonin transporter (SERT) noncompetitively by decreasing *V_max_* with little change in *K_m_* for its fluorescence substrate. In addition, treatment with lignan glycosides did not alter total and cell surface expression levels of the transporter protein. The two compounds decreased the accessibility of a cysteine residue placed in the extracellular substrate permeation pathway by inducing a conformational shift toward an outward-closed state of SERT. These results are consistent with molecular docking for the association of the lignan glycosides to the allosteric site in SERT. The present work supports the proposal that these compounds act on SERT by a novel underlying mechanism of action different from that of conventional antidepressant drugs.

## 1. Introduction

*Albizia julibrissin* Durazz., a leguminous deciduous shrub, is one of the most common herbs used for depression and anxiety treatment in East Asia. Its dried flowers or bark are generally processed for medicinal purposes. The main ingredients in *Albizia Julibrissin* Durazz. include triterpenoids, lignans, flavonoids, saponins, and sterols [1]. Preclinical studies showed that these ingredients exhibit a broad array of pharmacological activities ranging from antidepressant and anxiolytic [2,3,4,5], anti-inflammatory [6,7], antioxidant [8], and antitumor [9,10], to the enhancement of immunological function [11]. Their molecular mechanisms of action, however, are not understood.

Serotonin transporter (SERT) is a presynaptic plasma membrane protein responsible for 5-hydroxytryptamine (5-HT) reuptake after its release by serotonergic neurons [12,13]. SERT is the molecular target for antidepressants, such as selective serotonin reuptake inhibitors (SSRIs) including fluoxetine and imipramine. SERT belongs to the large transporter family called neurotransmitter sodium symporters (NSS). Together with transporters for dopamine and norepinephrine (DAT and NET), SERT is a member of a subgroup of transporters in the NSS family that symport biogenic amines with Na^+^ and Cl^−^ ions [14,15,16,17,18].

Serotonin–norepinephrine reuptake inhibitors (SNRIs) that are potent inhibitors of both SERT and NET, such as duloxetine [19] and venlafaxine [20], were approved as antidepressants or anxiolytics in clinical practice. Triple reuptake inhibitors (TRIs) concomitantly inhibit SERT, DAT, and NET. A TRI, nefazodone [21], was also approved as an antidepressant, but it was later withdrawn from the market due to its rare incidence of severe liver damage [22,23].

The transport of monoamines is proposed to interconvert two conformations of the monoamine transporters: outward-facing conformation that binds extracellular substrates and ions, and inward-facing conformation that releases substrates and ions to the cytoplasm [24,25,26,27,28,29,30]. Three-dimensional structures of DAT and SERT bound with their specific inhibitors were recently resolved [31,32,33,34,35,36], and provided structural insights into the molecular basis for antidepressant action on the monoamine neurotransmitter transporters. In these high-resolution structures, antidepressant molecules occupied the central binding cavity and thus competitively inhibited the conformational conversion required for the monoamine transport.

We previously demonstrated that a natural alkaloid, ibogaine, noncompetitively inhibited SERT by stabilizing the inward-facing conformation of the transporter [37,38]. Ibogaine differs from SSRIs, which competitively inhibit SERT by restraining SERT in an outward-facing conformation. Recently reported cryo-electron microscopy structures of SERT-ibogaine complexes uncovered the ibogaine binding site and mechanism of ibogaine inhibition [39]. These works shifted our efforts in developing antidepressants toward novel agents that target the conformation of monoamine transporters, which is different from the action of conventional antidepressant drugs.

We present the separation and identification of bioactive compounds from *Albizia julibrissin* Durazz. with antidepressant and anxiolytic properties. We monitored their effects on SERT activity by measuring the inhibition of a SERT fluorescent substrate uptake throughout the separation procedure. Two lignan glucosides were identified to be potent inhibitors of SERT. In addition, we used kinetic measurement and site-directed chemical modification to understand the molecular mechanism of action by which these compounds inhibit substrate transport by SERT. Furthermore, we examined the effects of these compounds on DAT and NET. Lastly, we conducted molecular docking of the lignan glycosides to the allosteric site in SERT.

## 2. Results

### 2.1. Separation and Identification of Bioactive Compounds Inhibiting SERT Activity

**Figure 1:**
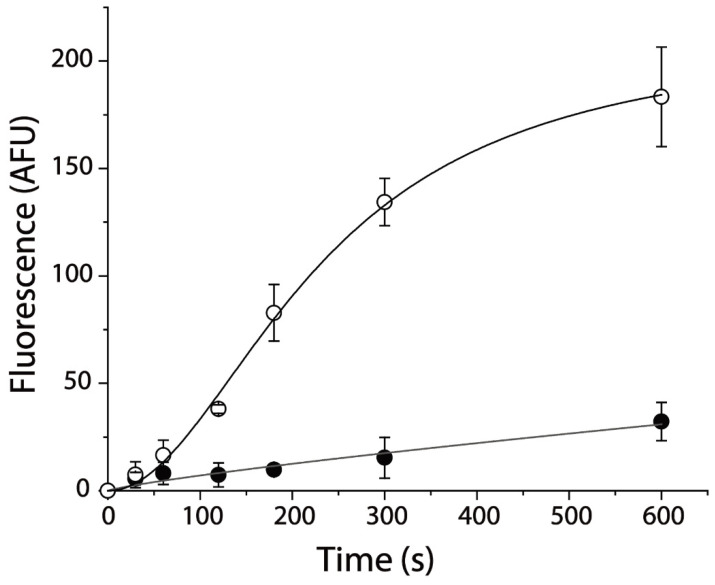
APP^+^ influx inhibited by specific inhibitor of hSERT, fluoxetine. Cells expressing hSERT incubated with APP^+^ in the absence (○) or presence (●) of 10 μM fluoxetine at room temperature for the indicated time. After 3 × rapid washing, APP^+^ fluorescence in the cells was measured as described in Section 4. Graph shows representative experiment with APP^+^ influx expressed as fluorescence (AFU). All error bars shown represent SDs from triplicate measurements. Experiment was repeated twice more with similar results.

To monitor the effects of *Albizia julibrissin* Durazz. extracts on SERT, a fluorescent substrate, 4-[4-(dimethylamino) phenyl]-1-methylpyridinium (APP^+^), was used for measuring the inhibitory potency of various constituents on its uptake. APP^+^ has emerged as a powerful tool to fluorometrically examine SERT transport in various aspects [40,41,42,43]. As shown in Figure 1, APP^+^ exhibited a superior fluorescence uptake in hSERT-expressing cells, which was inhibited by an SSRI, fluoxetine, suggesting that APP^+^ specifically monitors hSERT activity. APP^+^ uptake increased time-dependently and slowly reached saturation after 5 min reaction. At 5 min, approximately 90% of APP^+^ was blocked by preincubation with 10 μM fluoxetine, and residual fluorescence was considered to be nonspecific uptake by the cells.

**Figure 2:**
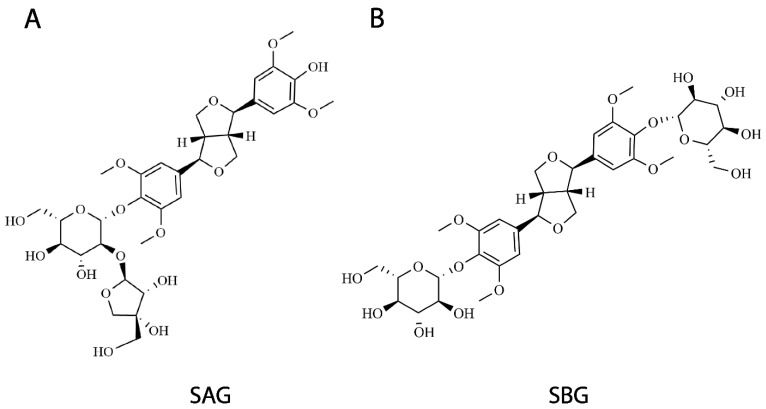
Structures of two lignan glycosides isolated in this study. Total extracts of *Albizia julibrissin* Durazz. successively separated by nonpolar macroporous adsorption, high-speed countercurrent chromatography, and preparative chromatography, and inhibitory effects of all fractions on hSERT activity were monitored as described in Section 4. Two compounds, (**A**) SAG and (**B**) SBG, with strongest potency antagonizing hSERT activity identified by structural analyses.

We examined the inhibitory effects of all fractions in the separation of *Albizia julibrissin* Durazz. on hSERT activity, and performed further separation on the fractions with strong potency antagonizing APP^+^ uptake according to their *K_i_* values. Two compounds that exerted the most significant effects on hSERT activity were isolated by a separation procedure with three chromatographic steps (Figure 2 and Figure S1) and subjected to the further structural identification. To characterize the two compounds, we performed spectral analyses of high-resolution mass spectrometry (MS), ^1^H-NMR (Figure S2), ^13^C-NMR (Figure S3), IR spectroscopy (Figure S4), circular-dichroism (CD) spectroscopy (Figure S5), and optical activity. Compound 1 or 2 showed an [M-H]^−^ ion peak at *m*/*z* 711.2510 or 741.2720 in its MS spectra, and a negative [α]_D_ value, respectively. In comparison with the spectral and optical characteristics of known compounds isolated from *Albizia julibrissin* Durazz. and other herbs, the two compounds were identified to be (-)-syringaresinol-4-O-β-D-apiofuranosyl-(1→2)-β-D-glucopyranoside (SAG) and (-)-syringaresinol-4,4′-bis-O-β-D-glucopyranoside (SBG), which had been reported [44,45,46,47,48,49]. The two compounds belong to lignan glycosides and share the same parental structure, (-)-syringaresinol (Figure 2).

### 2.2. Lignan Glycosides Noncompetitively Inhibited hSERT Activity

**Figure 3:**
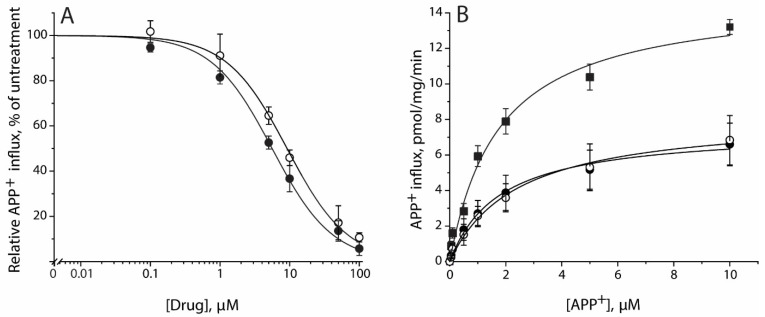
Inhibition of APP^+^ transport by SAG and SBG. (**A**) Inhibition of APP^+^ influx by SAG and SBG. APP^+^ influx into the cells expressing hSERT measured as described under Section 4 using 2 μM APP^+^ in the presence of indicated concentrations of SAG (●) or SBG (○). Control APP^+^ influx rate in inhibitor absence was 7.82 ± 1.12 pmol/min/mg. Nonspecific uptake measured in the presence of 10 μM fluoxetine and subtracted to give values of APP^+^ influx. Graph shows representative experiment, with APP^+^ influx expressed as a percentage of that measured in inhibitor absence. All error bars represent SDs from triplicate measurements. Experiment was repeated twice more with similar results. *K_i_* values for SAG and SBG were 5.25 ± 0.30 and 8.51 ± 0.50 μM, respectively. These calculated *K_i_* values represent mean ± SEM of three experiments with triplicate measurements in each experiment. (**B**) Noncompetitive inhibition of APP^+^ transport by SAG or SBG. APP^+^ influx into the cells expressing hSERT was measured as described under Section 4 using indicated concentration of APP^+^ in the absence (■) or presence of 5 μM SAG (●) or 8 μM SBG (○). Nonspecific uptake measured in the presence of 10 μM fluoxetine and subtracted to give shown values. Graph shows a representative experiment. All error bars represent SDs from triplicate measurements. The experiment was repeated twice more with similar results. *K_m_* and *V_max_* values represent mean ± SEM of three experiments with triplicate measurements in each experiment.

Lignan glycoside SAG or SBG inhibited APP^+^ uptake by the cells expressing hSERT. Data in Figure 3A demonstrate that APP^+^ transport was inhibited by SAG or SBG with a *K_i_* value of 5.25 ± 0.30 or 8.51 ± 0.51 μM, respectively (Table 1).

Lignan glycosides noncompetitively inhibited APP^+^ transport. Figure 3B shows that the simultaneous addition of SAG or SBG with APP^+^ significantly decreased transport *V_max,_* and there was little change in *K_m_* for APP^+^. *K_m_* values were 2.04 ± 0.24 μM in the control and 2.12 ± 0.53 μM or 2.73 ± 0.70 μM in the presence of SAG or SBG. *V_max_* values were 23.71 ± 1.98 pmol/min/mg for the control, and 11.26 ± 0.96 pmol/min/mg or 12.08 ± 1.15 pmol/min/mg for 5 μM SAG or 8 μM SAB, respectively. In separate experiments, SAG or SBG preincubation with cells for 5 min had no effect on the parameters of inhibition (data not shown).

### 2.3. Both SAG and SBG Inhibited APP^+^ Intracellular Accumulation

**Figure 4:**
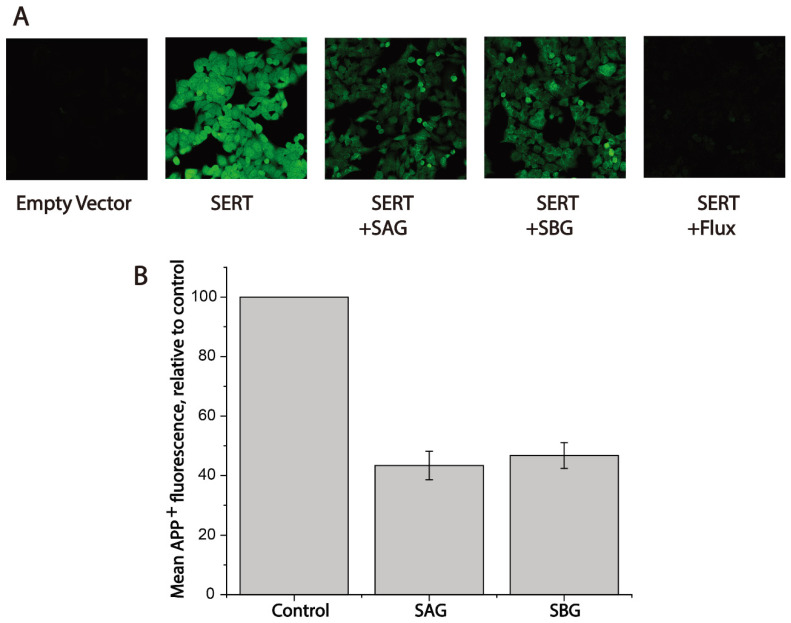
Fluorescence image analysis for inhibition of APP^+^ uptake by SAG and SBG. Cells transfected with hSERT or empty vector were wet-mounted on polylysine-coated glass slides and incubated with APP^+^ in the absence or presence of SAG (5 μM), SBG (8 μM), or fluoxetine (10 μM) for 5 min. After 3 × washing, accumulated APP^+^ fluorescence images were acquired and counted as described under Section 4. (**A**) Confocal fluorescence images parallelly acquired from one experiment. Experiment was repeated twice more with similar results. (**B**) Quantitative analysis for inhibition of APP^+^ uptake by SAG and SBG. In each experiment, at least ten cells were randomly selected for quantitative analysis (n ≥ 10). Accumulated APP^+^ fluorescence within cells counted using Zen Blue software and normalized to cell areas. Nonspecific uptake measured in the presence of 10 μM fluoxetine and subtracted when APP^+^ uptake was calculated. Graph is a combination of three experiments. All error bars represent the SEM.

To conform the inhibitory effects of SAG or SBG on APP^+^ transport, we performed fluorescent image analyses (Figure 4). Following exposure to 2 μM APP^+^ for 5 min, cells expressing hSERT exhibited bright intracellular fluorescence signal; conversely, hardly any APP^+^ signal was observed in the cells transfected with empty vector (Figure 4A). A hSERT specific inhibitor fluoxetine (10 μM) diminished the APP^+^ signal by more than 90%. Treatment with SAG or SBG significantly reduced APP^+^ intracellular accumulation, suggesting that lignan glycosides exerted their effect on hSERT by inhibiting APP^+^ transport. In addition, the intracellular fluorescence intensity of APP^+^ under drug treatments was measured and compared with the value without treatment. As shown in Figure 4B, SAG or SBG at its *K_i_* concentration inhibited APP^+^ accumulation by 40%–50%, which is consistent with the measurements using a microplate reader.

### 2.4. Both SAG and SBG Did Not Alter hSERT Expression

**Figure 5:**
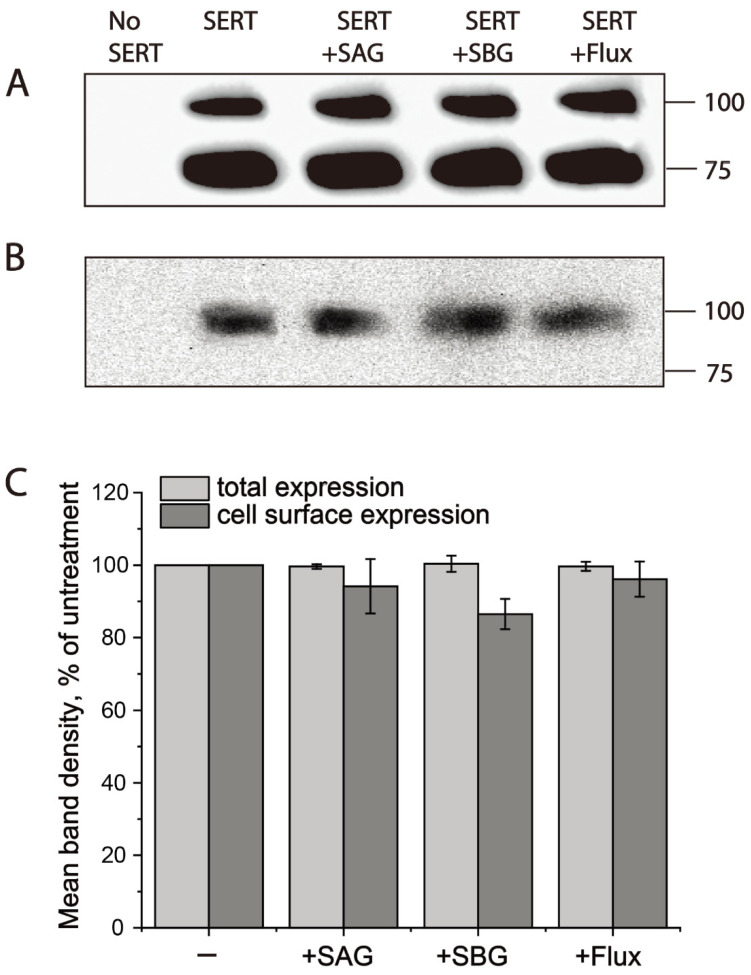
Both SAG and SBG did not alter hSERT expression. Cells expressing hSERT-Flag treated without or with SAG (2 × *K_i_*, 10 μM), SBG (2 × *K_i_*, 16 μM), or fluoxetine (10 μM) at room temperature for 10 min. After being labeled with NHS-SS-biotin at 4 °C for 30 min, cells were lysed with lysis buffer. Small portion of lysates analyzed for total hSERT-Flag expression. Cell-surface labelled proteins in the residual solution were captured with streptavidin–agarose and subjected to Western blotting analysis for hSERT-Flag cell surface expression, as described in Section 4. (**A**) Representative blot of total hSERT-Flag expression. (**B**) Representative blot of hSERT-Flag cell surface expression. (**C**) Quantitative analysis for effects of SAG and SBG on hSERT-Flag expression. Graph is a combination of three experiments. All error bars represent SEM.

To see if SAG or SBG changes hSERT expression, we measured total and cell surface expression levels of hSERT with or without drug treatments. Total cell lysates were used for measuring total hSERT expression, while cell surface expression of hSERT was determined by using cell surface biotinylation of plasma membrane proteins. As shown in Figure 5, treatment with SAG or SBG at its 2 × *K_i_* concentration did not alter either the total (Figure 5A,C) or cell surface (Figure 5B,C) expression level of hSERT, suggesting that SAG or SBG exerted its effects on hSERT activity by inhibiting hSERT catalytic function.

### 2.5. SAG or SBG Differed in Influence on hSERT Conformation from Fluoxetine

To examine whether SAG or SBG influences hSERT conformation, we utilized an assay based on the accessibility of a cysteine residue to determine their effects on the reactivity of the strategically positioned cysteine residue in the extracellular substrate permeation pathway (Y107C). We previously demonstrated that this cysteine reacts with the membrane-impermeant cysteine reagent 2-(trimethylammonium)ethyl methanethiosulfonate bromide (MTSET) more when the extracellular pathway is open, and less when the pathway is closed. These measurements of reactivity depend on the ability of cysteine reagents such as MTSET to inactivate SERT transport activity by an allosteric mechanism [50]. We proposed that, by modifying a cysteine residue such as Y107C in the extracellular permeation pathway, MTSET prevented the pathway closing, thus leading to the inactivation of APP^+^ transport.

**Figure 6:**
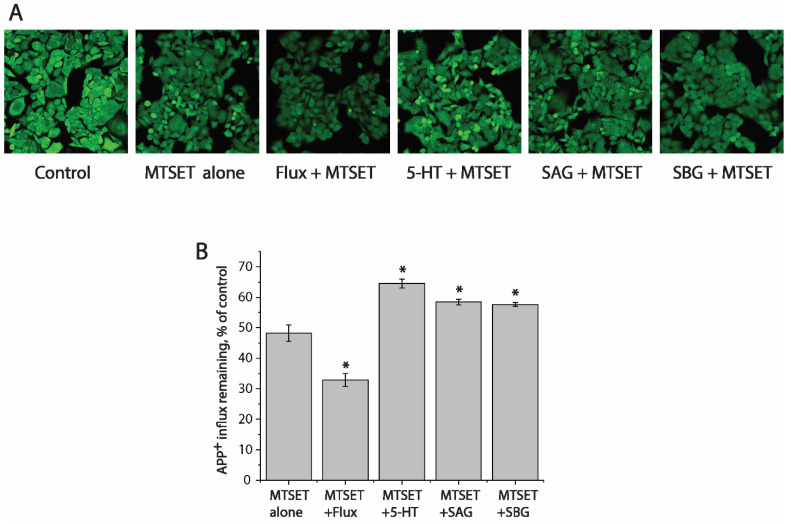
Effects of SAG and SBG on SERT conformation. (**A**) Representative images of accumulated APP^+^ in cells expressing Y107C mutant after treatment without (control) or with MTSET in the absence (MTSET alone) or presence of 10 μM fluoxetine (MTSET + flux), 10 μM SAG (MTSET + SAG), or 16 μM SBG (MTSET + SBG). The experiment was repeated twice more with similar results. (**B**) Quantitative analysis for APP^+^ influx after treatment with indicated drugs in the absence or presence of MTSET, expressed as a percentage of that measured in the absence of MTSET (control). In each experiment, at least ten cells were randomly selected for quantitative analysis. Accumulated APP^+^ fluorescence was counted and normalized to the cell areas. All error bars represent the SEM (n = 3). Asterisks indicate statistically significant changes (*p* < 0.05) in the accumulated APP^+^ fluorescence compared with the MTSET alone using Student’s *t* test.

We first determined a concentration of MTSET (0.01 mM) that inactivated ~50% of APP^+^ transport by SERT mutant Y107C in a 15 min incubation (Figure 6A,B, second image from left and second column from left, respectively). In these experiments to examine the effects of various ligands on SERT conformation, we incubated cells expressing Y107C with the indicated ligands and 0.01 mM MTSET. At the end of this incubation, cells were washed free of MTSET and ligands into KRH buffer containing NaCl and APP^+^. Altered ligand addition, therefore, was present only during the incubation with MTSET and not during the transport measurements. In this study, we measured the ability of ligands to influence the reactivity of Y107C with MTSET (Figure 6).

Competitive inhibitor fluoxetine, which stabilizes the outward-facing conformation of SERT [51], markedly increased the reactivity of Y107C promoting its inactivation by MTSET (Figure 6A,B, third image and second column from the left, respectively). In contrast, substrate 5-HT significantly decreased the reactivity of Y107C protecting it from inactivation (Figure 6A,B fourth image from the left and third column from right, respectively), consistent with our previous observation that 5-HT induced conformational conversion from outward-facing to inward-facing [15,17,52]. On the other hand, SAG or SBG exhibited potency to protect Y107C from MTSET inactivation, as compared to treatment with MTSET alone (Figure 6A, first and second images from the right, and Figure 6B, first and second columns from the right), indicating these lignan glycosides acted on SERT differently than fluoxetine did.

As a control experiment, we also examined the effect of MTSET on APP^+^ uptake by a cystine-less mutant, C109A, which lacks reactive cysteine residues on the extracellular surface of the transport protein. MTSET at the highest tested concentration (1 mM) had little effect on SERT activity (data not shown). Moreover, the addition of various ligands to cells expressing the C109A mutant did not alter its MTSET insensitivity (data not shown), consistently with previous measurements by using a radioactive substrate [53,54,55].

### 2.6. Both SAG and SBG Weakly Inhibited DAT and NET

To examine whether SAG or SBG inhibits DAT or NET, we performed transport assays in the presence of the lignan glycosides by using a fluorescence substrate for DAT and NET, 4-(4-(dimethylamino) styryl)-N-methylpyridinium (ASP^+^) [56,57,58]. SAG or SBG weakly inhibited ASP^+^ uptake into the cells expressing hDAT or hNET. As shown in Table 1, *K_i_* values of SAG and SBG for DAT and NET were ~20 to ~30 μM without a preference toward DAT or NET. In contrast, SAG or SBG exhibited a 3–4-fold more potency in inhibiting SERT than that of DAT and NET (Table 1).

### 2.7. Both SAG and SBG Did Not Exhibit Toxicity on Cell Proliferation

To see whether these lignan glycosides produce a toxic effect on cell proliferation, we conducted cytotoxicity assays by using a cell counting kit-8 (CCK-8). At the highest tested concentration (1 mM), both SAG and SBG had little effect on cell viability in cell proliferation (data not shown).

### 2.8. hSERT Possesses an Allosteric Binding Site for SAG or SBG

**Figure 7:**
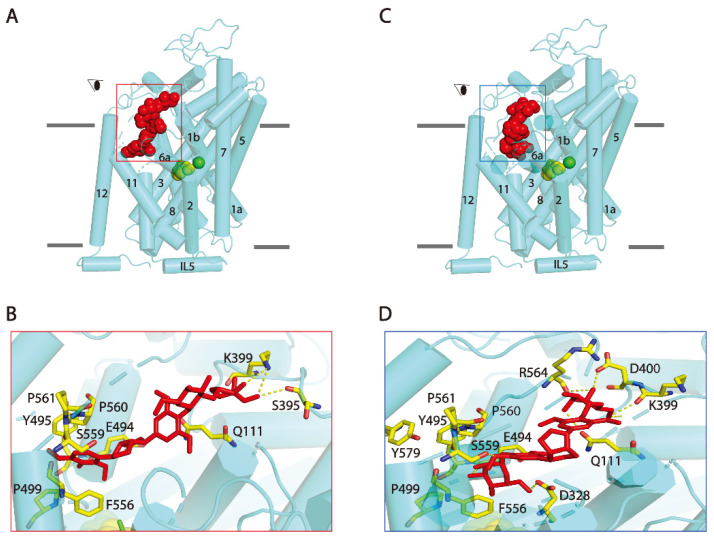
Molecular docking of SERT with SAG and SBG. Overall views of (**A**) SERT:SAG:5-HT and (**C**) SERT:SBG:5-HT complexes in cartoon representation. SAG (red) and 5-HT in central binding site (green and yellow) depicted as spheres. Separate green sphere represents Cl^−^ ion. The eye represents the angle views depicted in (**B**) and (**D**), respectively. (**B**,**D**) Close-ups of proposed SAG or SBG binding in the S2 site. Residues that interacted with SAG or SBG are annotated and shown in yellow sticks. IL: intracellular loop.

Both SAG and SBG noncompetitively inhibited SERT, suggesting that the two lignan glycosides do not bind to the central binding site (S1) in hSERT. Recent cryo-electron microscopy structures of hSERT revealed an allosteric site (S2) formed by an aromatic pocket positioned in the scaffold domain in the extracellular vestibule [36]. To examine the possibility that SAG or SBG binds to the S2 site, we conducted the molecular docking of hSERT-SAG or hSERT-SBG on a cryo-electron microscopy structure of hSERT in an outward-occluded state. Our analysis showed that both SAG and SAG fit well within the S2 binding pocket (Figure 7). SAG binding is mainly formed by side chain residues in TM1, 10, 11, 12, and extracellular loop (EL) 4a. Residues that interact with SAG include Gln111 inTM1, Glu494, Tyr495, Pro499 in TM 10, Phe556, Ser559 in TM11, Tyr579 in TM12, and Ser395, Lys399 in EL4a, of which Ser395, Lys399 and Glu494 form H-bonds with apiofuranosyl and diepoxylignane of SAG, respectively (Figure 7B). On the other hand, SBG adopts a bent conformation in the S2 site and interacts with Gln111 in TM1, Asp328 in TM6a, Lys399, Asp400 in EL4a, Glu494 in TM10, Phe556, Ser559, Arg564 in TM11. The two glucopyranosyl of SBG with Gln111, Asp328, Lys399, Asp400, Ser559, and Arg564 form 6 H-bonds in the S2 site, respectively (Figure 7D).

## 3. Discussion

This work isolated and identified two natural lignan glycoside compounds that possess potency in antagonizing SERT transport. The evidence presented here supports the proposal that lignan glycosides directly bind to the transport protein, presumably to the allosteric S2 site, thus noncompetitively inhibiting SERT activity by blocking essential conformational conversions for substrate transport. Our data showed that the (i) addition of SAG or SBG decreased *V_max_* with little change in the *K_m_* for the substrate (Figure 3), (ii) treatment with SAG or SBG did not alter total and cell surface expression levels of SERT (Figure 5), and (iii) SAG or SBG induced a conformational change in SERT toward an outward-closed state (Figure 6). All biochemical evidence is supported by molecular docking for the association of SAG or SBG to the S2 site in SERT (Figure 7).

Lignan glycosides exert their inhibitory effects on hSERT differently from conventional antidepressant drugs. Tricyclic antidepressants and SSRIs bind to the central binding site (S1), thus competitively inhibiting SERT activity by stabilizing the outward-facing conformation of the transport protein [59,60,61]. We previously identified a position on TM1 in the extracellular substrate permeation pathway of SERT that became more reactive in the presence of antidepressant drugs, such as fluoxetine, and less reactive in the presence of the substrate, 5-HT [37]. In this study, we monitored conformational changes of the extracellular pathway by employing a cysteine mutant at this position (Y107C) in response to various ligand treatments. Consistent with our previous observation, fluoxetine increased Y107C reactivity with MTSET, resulting in a significantly decreased APP^+^ uptake (Figure 6). In contrast, the lignan glycosides, SAG and SBG, prevented Y107C from MTSET modification, thus markedly increased APP^+^ uptake, in comparison with treatment with MTEST alone (Figure 6). Previous studies on the leucine transporter, a bacterial homologue of the NSS family, showed that the substrate occupancy or mutations of the S2 site caused profound effects on conformational changes of the transporter protein [25,62]. In agreement with these results, we show here that SAG or SBG binding shifts SERT conformation toward an outward-closed state. Importantly, our data indicated that these lignan compounds influence SERT conformation differently from the conventional antidepressant drugs (Figure 6).

An earlier study showed that administration of SAG (3.6 mg/kg, 7 days, p.o.) produced the antidepressant and anxiolytic responses in acute restraint-stressed rat models; however, the molecular mechanism was not uncovered [63]. An increase in 5-HT level in synapse, which in turn stimulates 5-HT neurotransmission, is the most common approach for the effective treatment of depression and anxiety. Our results showed that SAG is a potent inhibitor of SERT, providing evidence to understand its molecular basis and mechanism of action as an antidepressant or anxiolytic drug. Although both SAG and SBG inhibit SERT activity, they also possess inhibitory effects on DAT and NET. The lack of specificity for SERT and NET increases our concerns about their addictive side effects caused by elevating synaptic concentrations of dopamine [64].

Depression is a popular and all-age-related mental illness, affecting approximately 10%–15% of the global population [65,66]. Depressive patients suffer greatly, and function poorly at work, study, and social activities. Severe depression can lead to disability and suicide. Conventional antidepressant drugs have many shortcomings, such as slow onset, low efficacy, and serious adverse effects, supporting the development of novel agents that exert a variety of pharmacological actions. All available antidepressant drugs competitively inhibit SERT by binding to the central binding site. In this work, we report two natural compounds inhibit SERT by a different underlying mechanism. While their potency and selectivity were lower than those of the synthesized antidepressant drugs, we expect that these compounds could become lead molecules for the development of novel therapeutic agents with a divergent mechanism of action.

## 4. Materials and Methods

### 4.1. Materials

The bark of *Albizia Julibrissin* Durazz. was obtained from Anhui Xiehecheng Pharmaceutical Co. Ltd., Anhui, China, (batch no. 19120704). HeLa cells (CCL-2) were from American Type Culture Collection. N-terminal His6 and C-terminal FLAG-tagged human SERT (hSERT), C109A, and Y107C/C109A expression constructs used in this study were described previously [37,67]. Expression plasmids for human DAT (hDAT) and human NET (hNET) in pcDNA3.1 were from the Dr. Rudnick lab (Yale School of Medicine). APP^+^, ASP^+^, protease inhibitor mixture cocktail, anti-Flag monoclonal M2 antibody, fluoxetine, GBR 12909, and desipramine were purchased from Sigma-Aldrich. EZ-Link™ NHS-SS-Biotin, streptavidin-agarose, Super Signal West Femto, lipofectamine 2000, and Micro BCA protein assay reagent kit were obtained from Thermo Fisher Scientific. MTSET was purchased from Biotium. Cell counting kit-8 (CCK-8) was from Dojindo (Shanghai, China). All other reagents were of analytical grade.

### 4.2. Separation and Identification of Bioactive Compounds

The dried bark of *Albizia Julibrissin* Durazz. was refluxed in a boiling 70% ethanol solvent for 2 h, and total extracts were first separated by a D101 nonpolar macroporous adsorption resin with 60% ethanol as a mobile phase. The eluate was concentrated and then subjected to the second separation using high-speed countercurrent chromatography (Tauto TBE-300B, Tongtian, Shanghai, China) with a solvent system composed of n-butanol, water, ethyl acetate, acetic acid, methanol (30:30:10:1:4). Fractions that showed high potency in antagonizing APP^+^ uptake were further separated by preparative chromatography (LC-6AD, Shimadzu, Kyoto, Japan) under the following conditions: column, Shimadzu EW0291; mobile phase, acetonitrile: water (0:100→50:50, 30 min); flow rate, 8 mL/min; temperature, 23 °C; injection volume, 400 μL; detection wavelength, 210 nm.

Two fractions that each contained a single peak in preparative chromatography were collected and analyzed for their purity by high-performance liquid chromatography (Agilent 1260 LC, Agilent, Santa Clara, USA) under the following conditions: column, AgilentZORBAXSB-C18 (4.6 × 250 mm); mobile phase, acetonitrile:0.04% phosphoric acid (5:95→95:5, 30 min); flow rate, 1.0 mL/min; temperature, 30 °C; injection volume, 10 μL; detection wavelength, 210 nm. On the basis of their HPLC chromatograms, each fraction contained a single compound with more than 95% purity (Figure S1). The amounts of the two compounds in the dried bark of *Albizia julibrissin* Durazz. were estimated by HPLC to be 0.34% for Compound **1,** and 0.04% for Compound **2**. The two compounds were then applied to spectral analyses to identify their structures. The major spectral characteristics of the two compounds are shown below.

Compound **1**, white amorphous powder, [α]^24^_D_: −3.8 °C (MeOH), MS: [M-H]^−^ ion peak at *m*/*z* 711.2510 (calculated value for C_33_H_43_O_17_^−^, 711.2532); CD: -0.69535 at 206 nm; ^1^H-NMR δ (300 MHZ, methanol-d4): 6.74 (2H, H-2, H-6), 6.68 (2H, H-2′, H-6′), 4.89 (1H, H-1″), 4.79 (1H, H-7′), 4.73 (1H, H-7), 4.31 (2H, H-9a, 9′a), 4.30 (2H, H-9b, 9′b), 3.22 (1H, H-8′), 3.15 (1H, H-8), 3.88 (6H, s, 3, 5 -OCH_3_), 3.86 (6H, s, 3′, 5′-OCH_3_); ^13^C-NMR (75 MHZ, methanol-d): 153.0 (C-3, C-5), 148.0 (C-3′, C-5′), 138.2 (C-4), 134.8(C-4′), 134.2 (C-1), 131.7 (C-1′), 104.4 (C-1″), 103.4 (C-2, C-6), 103.1 (C-2′, C-6′), 86.2 (C-7′), 85.8 (C-7), 77.0 (C-5″), 76.4 (C-3″), 74.3 (C-2″), 71.5 (C-9, 9′), 70.3 (C-4″), 61.2 (C-6″), 55.7 (-OCH_3_*2), 56.4 (-OCH_3_*2), 54.3 (C-8′), 53.7 (C-8); IR: 3353 (-OH), 2936 (CH_2_), 2881 (CH_3_), 1602 (aromatic C=C), 1511 (aromatic C=C), 1463 (CH_2_), 1331 (OH), 1227 (C-O-C), 1115 (C-O-C), 1060 (C-O-C), 823 (CH). The spectral features match well with the reported compound, (-)-SAG [44,45,46].

Compound **2**, white amorphous powder, [α]^24^_D_: −10.5 °C (MeOH); MS: [M-H]^−^ ion peak at *m*/*z* 741.2720 (calculated value for C_34_H_45_O_18_^−^, 741.2705); CD: −0.1046 at 206 nm; ^1^H-NMR δ (300 MHZ, methanol-d4): 6.62 (2H, H-2, H-6), 6.58 (2H, H-2′, H-6′), 5.40 (1H, H-1‴), 4.89 (1H, H-1″), 4.68 (1H, H-7′), 4.64 (1H, H-7), 4.20 (2H, H-9a, 9′a), 3.84 (2H, H-9b, 9′b), 3.77 (12H, s, 4*OCH_3_), 3.06 (2H, H-8, H-8′); ^13^C-NMR (75 MHZ, methanol-d): 153.2 (C-3′, C-5′), 148.0 (C-3, C-5), 137.8 (C-4′), 134.8 (C-4), 133.7 (C-1′), 131.7 (C-1), 109.0 (C-1‴), 103.4 (C-2, C-6), 103.1 (C-2′, C-6′), 101.3 (C-1″), 86.2 (C-7′), 85.8 (C-7), 79.5 (C-3‴), 77.3 (C-2″), 77.2 (C-2‴), 76.7 (C-3″), 76.6 (C-5″), 74.2 (C-4‴), 71.5 (C-9, 9′), 70.0 (C-4″), 65.0 (C-5‴), 61.2 (C-6″), 55.6 (-OCH_3_*2), 55.4 (-OCH_3_*2), 54.3 (C-8′), 54.1 (C-8); IR: 3367 (OH), 2929 (CH_2_), 2860 (CH2), 1595 (aromatic C=C), 1511 (aromatic C=C), 1463 (CH_2_), 1421 (CH_3_), 1372 (CH_3_), 1233 (C-O-C), 1136 (C-O-C), 1074 (C-O-C), 816 (CH). The spectral features are consistent with the reported compound, (-)-SBG [47,48,49].

### 4.3. Expression of hSERT, hDAT, and hNET

The used expression systems are described elsewhere [61]. HeLa cells were cultured in Dulbecco’s Modified Eagle’s Medium supplemented with 10% fetal bovine serum, 100 units/mL penicillin, and 100 μg/mL streptomycin at 37 °C in a humidified 5% CO_2_ incubator. Cells were plated in 96-, 12-, or 6-well culture plates and grown overnight. Cells at ~70% confluency were transfected with hSERT, hDAT, or hNET cDNA in pcDNA3.1 by lipofectamine 2000. Transfected cells were incubated for 24–30 h at 37 °C with 5% CO_2_ and then assayed for APP^+^ uptake measurements, APP^+^ fluorescence image acquisition, or hSERT biotinylation. Protein concentration was determined with the Micro BCA protein assay reagent kit.

### 4.4. APP^+^ or ASP^+^ Uptake Measurements

Experiments were performed at room temperature (22 °C). Transfected cells in 96-well plates were washed once with 100 μL of KRH buffer containing 20 mM HEPES, pH 7.4, 120 mM NaCl, 1.3 mM KCl, 2.2 mM CaCl_2_, 1.2 mM MgSO_4_, and 0.1% (*w*/*v*) glucose. APP^+^ or ASP^+^ influx was measured by adding 100 μL of KRH buffer containing 2 μM APP^+^ or ASP^+^ and incubating for 5 min at room temperature. Excess APP^+^ or ASP^+^ was then removed by rapid washing three times with KRH buffer. The extent of APP^+^ accumulated in the cells was determined with the Infinite 200 Pro Microplate Reader (Tecan, Grodig, Austria). The excitation wavelength for APP^+^ or ASP^+^ was 488 nm, while used emission filters were 525 nm for APP^+^ or 580 nm for ASP^+^. Nonspecific transport was measured in the presence of 10 μM fluoxetine, GBR 12909, or desipramine for hSERT, hDAT, or hNET and was subtracted to give APP^+^ or ASP^+^ influx, respectively.

### 4.5. Fluorescence Image Acquisition and Fluorescence Intensity Analysis

Transfected or parental cells were wet mounted on glass slides and applied for the indicated treatments. Images were acquired at 20× using the Zeiss LSM 900 confocal microscope with an excitation wavelength at 488 nm. Images were analyzed using Zen Blue software. Fluorescence intensity was counted and normalized to cell areas.

### 4.6. Cell Surface Biotinylation

Cell surface expression of hSERT-Flag was determined using the membrane-impermeant biotinylation reagent sulfo-NHS-SS-biotin as described previously [68]. Briefly, HeLa cells expressing hSERT-Flag were treated twice with NHS-SS-biotin for 20 min on ice. After labeling, the cells were rinsed with 100 mM glycine in phosphate-buffered saline (137 mM NaCl, 2.7 mM KCl, 4.3 mM Na_2_HPO_4_, and 1.4 mM KH_2_PO_4_, pH 7.3) for 20 min to quench excess biotinylation reagent. Cells were then lysed with lysis buffer (50 mM Tris-HCl, pH 7.5, 150 mM NaCl, 5 mM EDTA, 1% Triton X-100, 0.1% SDS, 0.2 mM phenylmethylsulfonyl fluoride, and 0.5% Sigma protease inhibitor mixture), and biotinylated proteins were captured using streptavidin-agarose in 3 h incubation at 4 °C with gentle agitation. Biotinylated proteins were eluted with 100 μL of SDS-PAGE sample buffer and applied to a 10% SDS-polyacrylamide gel and visualized by Western blotting. hSERT was detected using anti-FLAG M2 monoclonal antibody (1:1000) against the FLAG epitope tag at the C-terminus of hSERT. A horseradish peroxidase-conjugated antimouse IgG (1:10,000) was used to visualize the signal by Super Signal West Femto. The amount of surface expression was determined by quantitative luminescence imaging using a UVP Biochemi imaging system.

### 4.7. CCK-8 Cytotoxicity Assays

Confluent cells in a 96-well plate were added in 10 μL of CCK-8 solution and additional incubation continued at 37 °C in a 5% CO_2_ incubator for 4 h. Quantitation of cell viability in cell proliferation and cytotoxicity assays was performed by using the Infinite 200 Pro Microplate Reader according to the manufacturer’s protocol.

### 4.8. Molecular Docking

Molecular docking was carried out with Glide software in Schrödinger Suites v2021.2 on a cryo-electron microscopy structure of hSERT in an outward-occluded state (PDB ID, 7MGW, 3.5 Å). The 15B8 Fab (chains B and C) and ligands such as 5-HT in the S2 site, acetylglucosamine, cholesterol, pentane, heptane, decane, and dodecane present in the structure were removed. The template structure was then subjected to automated structure preparation using the Protein Preparation Wizard in order to optimize the hydrogen bonding network, conformation of bonds and energy constraints. Ligand preparations were preformed using Ligprep. The SAG or SBG molecule was input into Maestro and optimized for its conformation and energy in the OPLS4 force field [69]. Protonation states of the ligands were calculated using Epik at pH 7.0 ± 2.0. The S2 allosteric site in hSERT was defined with Grid Generation as the central docking site with docking length ≤ 20 Å for the ligands. Docking was performed using the Glide module of Schrödinger under a standard precision, with the ligands in flexible conformations. In the docking step, 20 poses for each ligand were generated using Van der Waals radius scaling of 0.8 for proteins and ligands. The ligands posing with the S2 residues of at least one atom within 5 Å were subject to conformational search and energy minimization. The refined SAG-hSERT or SBG-hSERT complexes were ranked by glide scores. The more negative the glide score was, the more favorable the ligand binding to the S2 residues. One pose with the lowest energy was exported into PyMOL for visualization.

### 4.9. Data Analysis

Nonlinear regression fits of experimental and calculated data were performed with Origin (OriginLab). Statistical analysis given was from multiple experiments. In the figures, data with error bars represent the mean ± S.D. from triplicate measurements. Asterisks indicate significance at the *p* < 0.05 level in the paired Student’s *t* tests.

## Figures and Tables

**Table 1:** *K_i_* values for SAG and SBG.

	*K_i_* for SAG (μM)	*K_i_* for SBG (μM)
SERT	5.25 ± 0.30	8.51 ± 0.50
DAT	20.37 ± 2.13	32.28 ± 2.85
NET	17.36 ± 1.94	30.05 ± 3.61

Cells expressing hSERT, hDAT or hNET were incubated with 2 μM APP^+^ for hSERT or 2 μM ASP^+^ for DAT and NET, in absence or presence of SAG or SBG at various concentrations in a range of 0–100 μM. Accumulated fluorescence in cells was counted as described in Section 4. As controls, *K_i_* values of fluoxetine, GBR12909, or desipramine for SERT, DAT, or NET were 0.69 ± 0.07, 0.87 ± 0.09, or 1.23 ± 0.11 μM, respectively. Nonspecific transport measured in the presence of 10 μM fluoxetine, GBR 12909, or desipramine for hSERT, hDAT, or hNET, and was subtracted to give APP^+^ or ASP^+^ influx, respectively. These calculated *K_i_* values represent mean ± SEM of three experiments with triplicate measurements in each experiment.

## Data Availability

Data sharing is contained in this article.

## Supplementary Materials

The following supporting information can be downloaded at: https://www.mdpi.com/article/10.3390/ph15030344/s1, Figure S1: HPLC profiles of SAG (A) and SBG (B). The two compounds obtained by a separation procedure under “Materials and Methods” were analyzed for their purity by HPLC with an Agilent ZORBAXSB-C18 column and acetonitrile/0.04% phosphoric acid as a mobile phase; Figure S2: ^1^H-NMR spectrum of SAG (A) and SBG (B). ^1^H-NMR spectroscopy of the two compounds was detected with the Bruker NMR spectrometer Avance III 400 according to the manufacturer’s manual.; Figure S3: ^13^C-NMR spectrum of SAG (A) and SBG (B). ^13^C-NMR spectroscopy of the two compounds was detected with the Bruker NMR spectrometer Avance III 400 according to the manufacturer’s manual.; Figure S4: IR spectrum of SAG (*A*) and SBG (*B*). IR spectroscopy of the two compounds was measured with the IR spectrometer BRUKER TENSOR II under the resolution of 4 cm^−1^. The sample or background scan time was set at 16 scans, respectively, and the concave rubber band was used as a baseline correction method.; Figure S5: CD spectrum of SAG (A) and SBG (B). CD spectroscopy of the two compounds was measured with the CD spectrometer Chirascan under the conditions: detector type, PMT; time per point, 0.5 s; pathlength, 10 mm; wavelength, 180–400 nm; step size, 1 nm; Bandwidth, 1 nm.
