# Supplementary material for: Two Lignan Glycosides from Albizia julibrissin Durazz. Noncompetitively Inhibit Serotonin Transporter
Source: Pharmaceuticals (Basel). 2022 Mar 11;15(3):344. doi: 10.3390/ph15030344 (PMC8954383; doi:10.3390/ph15030344)
Supplement: Supplementary file 1 [file pharmaceuticals-15-00344-s001.zip › pharmaceuticals-1597321-supplementary.pdf]

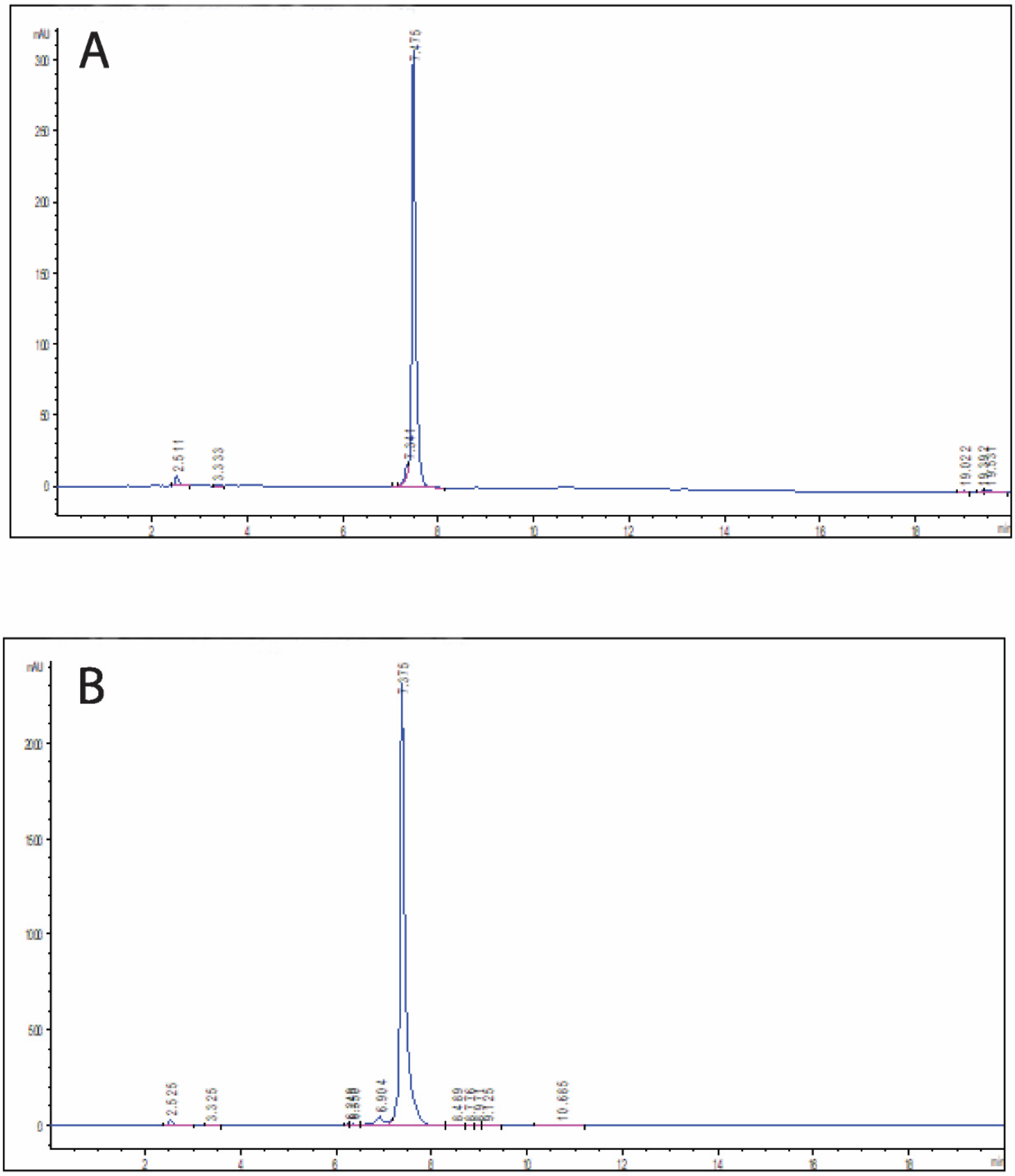

Figure S1. HPLC profiles of SAG (*A*) and SBG (*B*). The two compounds obtained by a separation procedure under "Materials and Methods" were analyzed for their purity by HPLC with a AgilentZORBAXSB-C18 column and acetonitrile/0.04% phosphoric acid as a mobile phase.

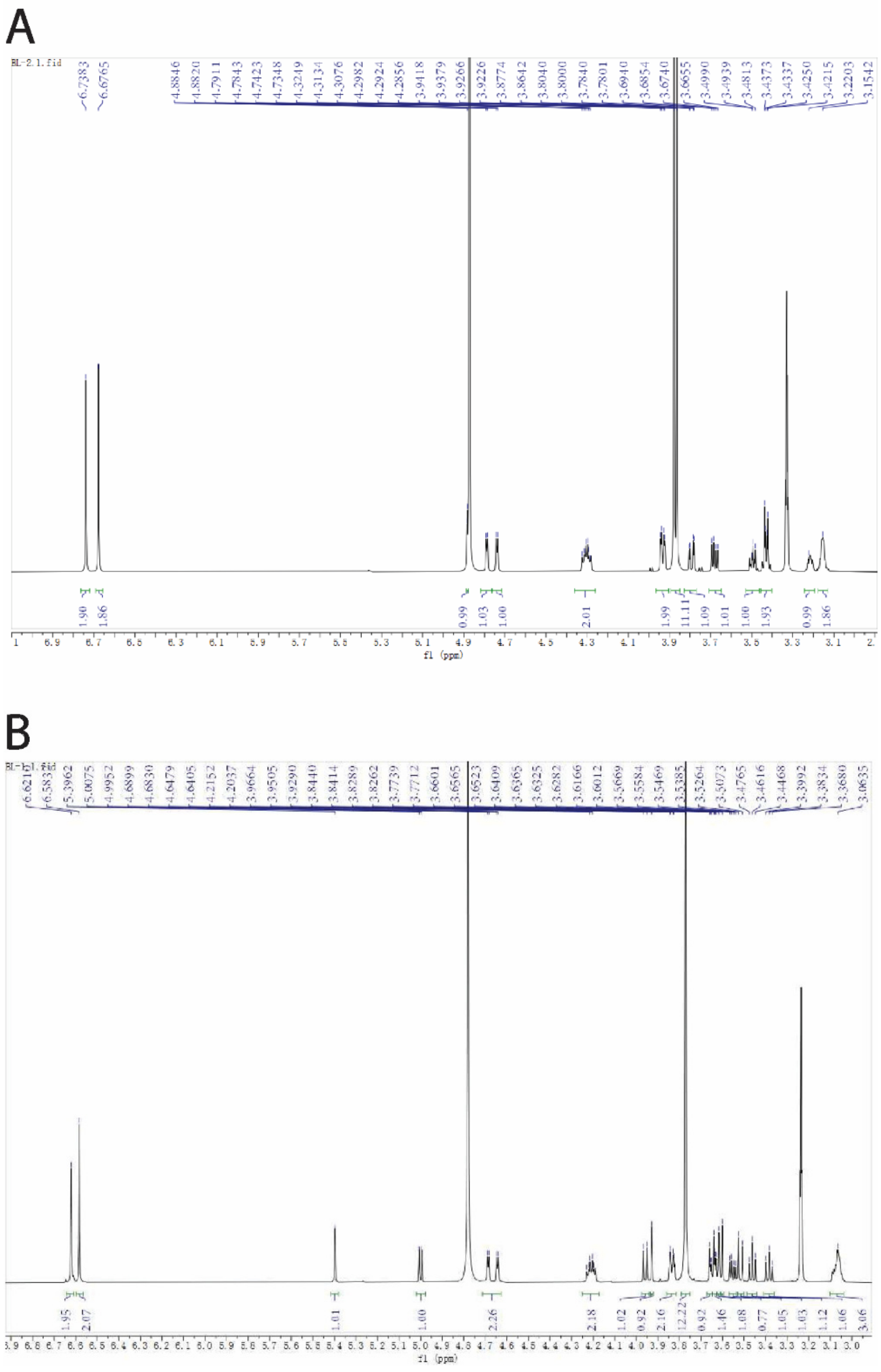

Figure S2. ¹H-NMR spectrum of SAG (*A*) and SBG (*B*). ¹H-NMR spectroscopy of the two compounds was detected with the Bruker NMR spectrometer Avance III 400 according to the manufacturer's manual.

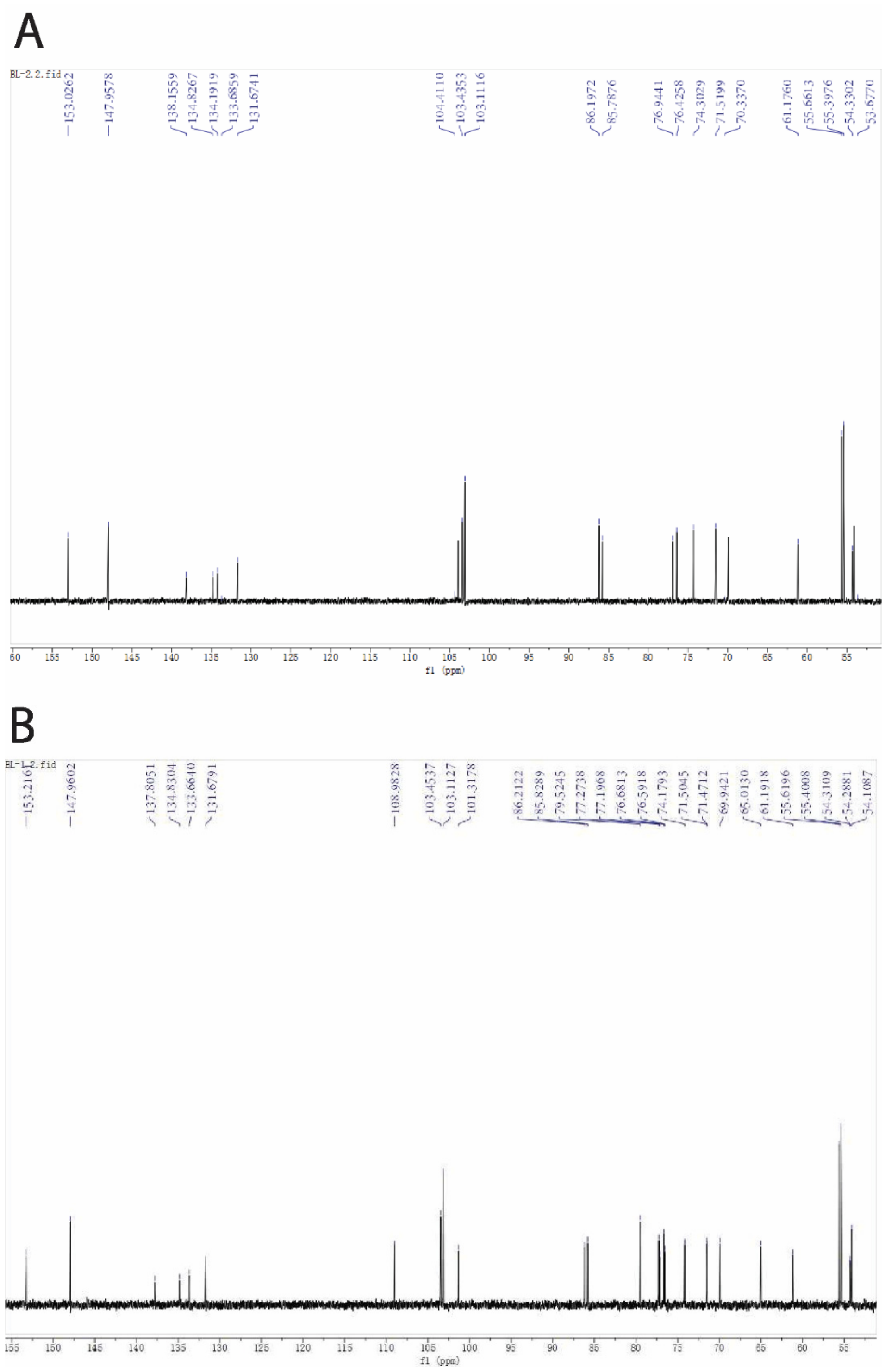

Figure S3. ^{13}C-NMR spectrum of SAG (A) and SBG (B). ^{13}C-NMR spectroscopy of the two compounds was detected with the Bruker NMR spectrometer Avance III 400 according to the manufacturer's manual.

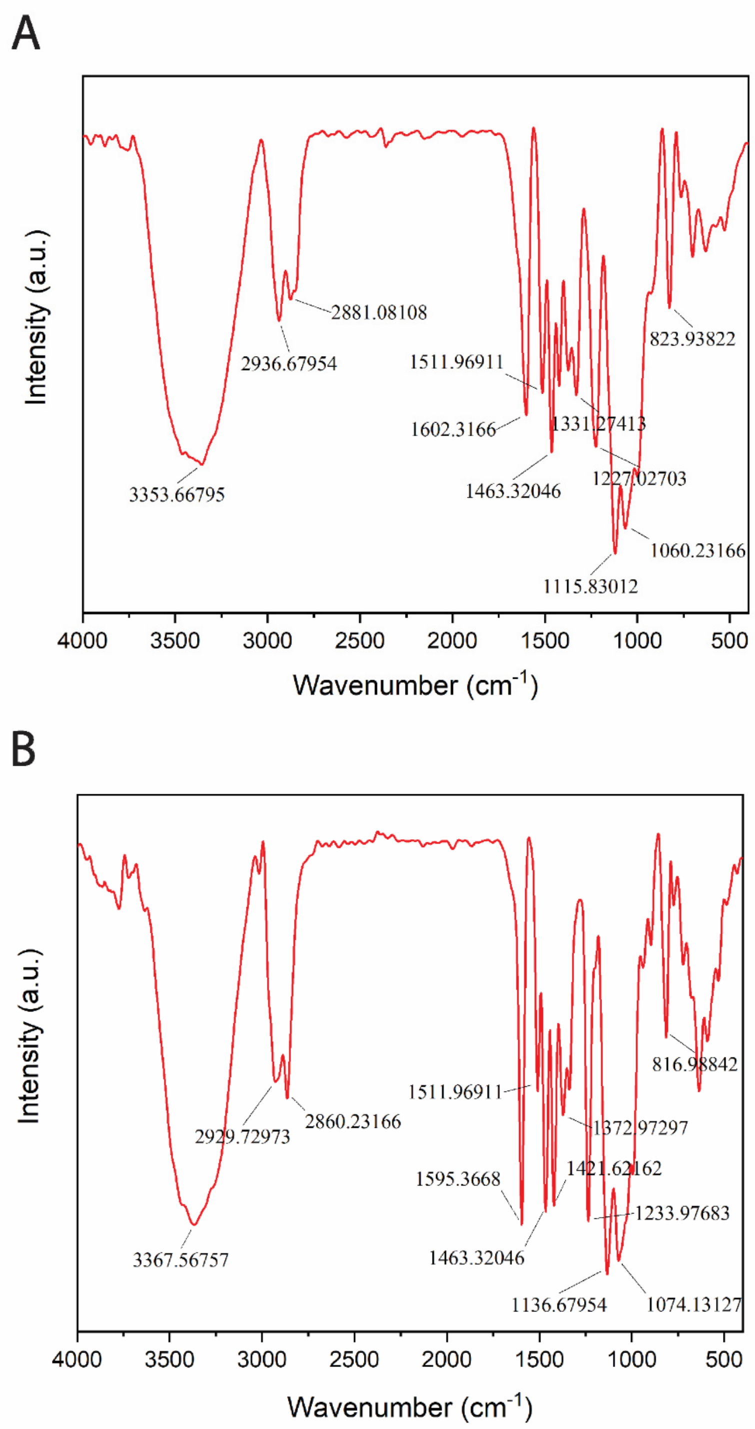

Figure S4. IR spectrum of SAG (*A*) and SBG (*B*). IR spectroscopy of the two compounds was measured with the IR spectrometer BRUKER TENSOR Ⅱ under the resolution of 4 cm^{-1}. The sample or background scan time was set at 16 scans, respectively, and the concave rubber band was used as a baseline correction method.

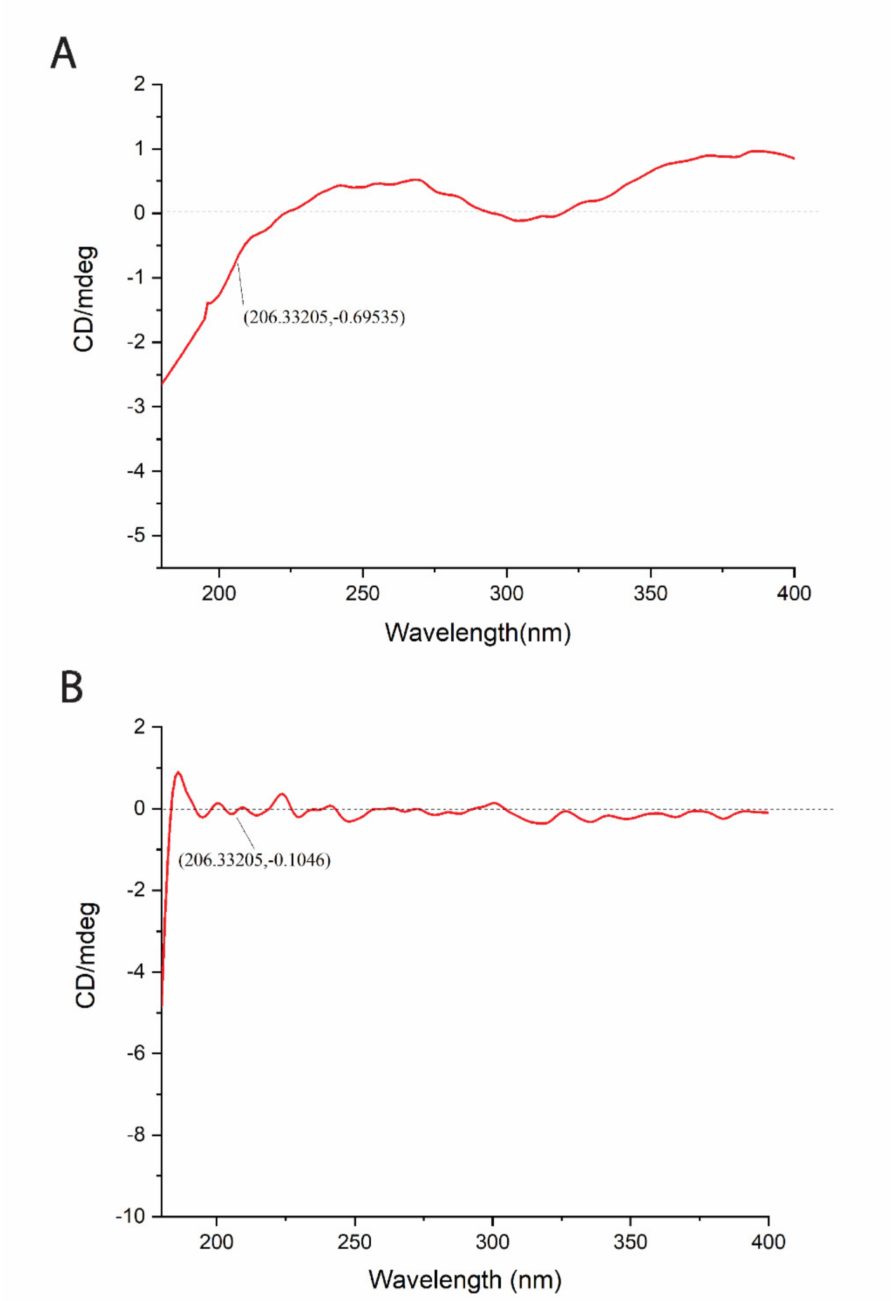

Figure S5. CD spectrum of SAG (A) and SBG (B). CD spectroscopy of the two compounds was measured with the CD spectrometer Chirascan under the conditions: detector type, PMT; time per point, 0.5 s; pathlength, 10 mm; wavelength, 180 – 400 nm; step size, 1 nm; Bandwidth, 1 nm.
